# Genomic versus phenotypic selection to improve corn borer resistance and grain yield in maize

**DOI:** 10.3389/fpls.2023.1162440

**Published:** 2023-07-07

**Authors:** Noemi Gesteiro, Bernardo Ordás, Ana Butrón, María de la Fuente, José Cruz Jiménez-Galindo, Luis Fernando Samayoa, Ana Cao, Rosa Ana Malvar

**Affiliations:** ^1^Mision Biologica de Galicia (CSIC), Pontevedra, Spain; ^2^Instituto Nacional de Investigaciones Forestales Agrícolas y Pecuarias (INIFAP), Chihuahua, Mexico; ^3^Department of Crop Science, North Carolina State University, Raleigh, NC, United States

**Keywords:** maize, stem borer, resistance, yield, genomic selection, phenotypic selection

## Abstract

**Introduction:**

The study of yield and resistance/tolerance to pest are related traits fundamental for maize breeding programs. Genomic selection (GS), which uses all marker information to calculate genomic breeding values, is presented as an emerging alternative to phenotypic and marker-assisted selections for improving complex traits controlled by many genes with small effects. Therefore, although phenotypic selection (PS) has been effective for increasing resistance and yield under high infestation with maize stem borers, higher genetic gains are expected to be obtained through GS based on the complex architecture of both traits. Our objective was to test whether GS is more effective than PS for improving resistance and/or tolerance to maize stem borers and grain yield.

**Methods:**

For this, we compared different selection programs based on phenotype and genotypic value for a single trait, resistance or yield, and for both traits together.

**Results and discussion:**

We obtained that GS achieved the highest genetic gain for yield, meanwhile phenotypic selection for yield was the program that achieved the highest reduction of tunnel length, but was ineffective for increasing yield. However, phenotypic or genomic selection for increased resistance may be more effective in improving both traits together; although the gains per cycle would be small for both traits.

## 1 Introduction

The Mediterranean Corn Borer (MCB), *Sesamia nonagrioides* Lefèbvre (Noctuidae), is a major pest of maize (*Zea mays* L.) in the Mediterranean region (Velasco et al., 2007; Jika et al., 2020). In Spain, MCB second and subsequent generations larvae cause the main damages inside maize stems by excavating longitudinal tunnels that destroy the pith and negatively affect plant development. Tunnels increase the susceptibility to plant lodging and interferes with assimilate movement to developing cobs, which reduces maize yields (Malvar et al., 2008). This has led to the search for varieties with increased natural resistance to MCB damage, which can be the basis for breeding programs aimed at releasing resistant and/or tolerant varieties (Butrón et al., 1999; Butrón et al., 2005; Revilla et al., 2007). Most studies have evaluated insect resistance in a wide sense, using the pith tunnel length produced by MCB under artificial infestation as the classifying criteria (Malvar et al., 1993; Butrón et al., 1999; Velasco et al., 1999a; Velasco et al., 1999b; Malvar et al., 2004; Velasco et al., 2004a; Butrón et al., 2005; Butrón et al., 2006; Malvar et al., 2007; Revilla et al., 2007). Once sources of resistance were identified (Butrón et al., 1999; Malvar et al., 2004), research was oriented to study trait inheritance (Cartea et al., 1999; Velasco et al., 2002; Velasco et al., 2004b; Butrón et al., 2009) and to develop the most suitable phenotypic selection programs to increase maize resistance and/or tolerance to MCB (Sandoya et al., 2008; Butrón et al., 2012; Ordás et al., 2012; Samayoa et al., 2012; Butrón et al., 2014).

Nevertheless, valuable genetic gains through phenotypic selection have been hindered by the unfavorable genetic correlation between tunnel length by MCB and yield (Sandoya et al., 2010; Butrón et al., 2012). Population yield was maintained in the first three cycles of phenotypic selection (PS) for reducing stem tunneling by MCB maintaining yield (Sandoya et al., 2008), but the yield decreased in the subsequent cycles (Butrón et al., 2012). In addition, the search for QTL for resistance to MCB damage and yield in segregating populations for both traits have rendered co-localized QTL for yield and resistance, indicating the presence of pleiotropism or linkage between genes contributing to the negative relationship between resistance and yield (Ordás et al., 2010; Jiménez-Galindo et al., 2017).

An alternative to PS could be Marker Assisted Selection (MAS) using markers linked to QTL with significant effects on resistance to stem tunneling by MCB but without adverse effects on yield (Ordás et al., 2010; Samayoa et al., 2019). However, commonly employed MAS strategies are not suitable for improving complex agronomic traits, in part, due to the lack of power to capture small effect loci that are often important in the inheritance of these traits since many minor genes are involved in MCB resistance, measured as reduced pith gallery length, and yield (Butrón et al., 2009; Butrón et al., 2012). For this reason, genomic selection (GS), which uses all marker information to calculate genomic estimated breeding values (GEBV), is presented as an emerging alternative to MAS for improving complex traits; genome-wide molecular marker coverage is able to capture both large and small effect QTL. It is also worth noting that genotyping and sequencing technology is advancing at an extremely rapid pace, with reducing cost. This coupled with continued advances in computational techniques to predict GEBV has great potential to increase accuracy at little or no additional cost; whereas phenotyping costs are stagnant or increasing. Therefore, GS would allow the design of selection programs with a higher genetic gain per year and a budget equivalent to that of a MAS program (Heffner et al., 2010; Zhang et al., 2017; Rice and Lipka, 2021). However, these predictions are based on simulation studies and there are few GS programs to validate such predictions (Heffner et al., 2010; Rice and Lipka, 2021). The assessment of genetic gain using GS has been exemplified in maize by rapid cycling recombination of biparental populations for drought tolerance (Beyene et al., 2015; Vivek et al., 2017),multiparent populations for grain yield under different moisture regimes (Zhang et al., 2017; Das et al., 2020) and resistance to *F. verticillioides* using a diverse set of tropical germplasm (Kuki et al., 2020), but no GS programs have been carried out to improve resistance to pests.

In this study, two selection cycles of six selection programs have been carried out to: 1) reduce the length of the tunnels produced by borers using the phenotypic value as selection criteria; 2) reduce the length of the tunnels using the GEBV; 3) improve yield using the phenotypic value; 4) yield improvement based on GEBV; 5) simultaneously improve both traits (tunnels and yield) using the phenotypic values of the index; and 6) improve yield and tunnels using GEBV of the index. The objective was to test whether SG, based on GEBV, is more effective than SP, based on phenotypic values, to improve resistance (short tunnels) using program 1 versus 2; tolerance (high yield under high pest pressure conditions) comparing programs 3 and 4, and/or tolerance and resistance simultaneously based on the comparison of programs 5 and 6.

## 2 Materials and methods

A population of F_6_ recombinant lines (RILs) was constructed from an F_2_ derived from the cross of two inbred lines (A637 × EP42) with contrasting tolerance to MCB. A637 is tolerant to MCB on the basis that it shows low yield losses caused by MCB damage, while EP42 is sensitive to MCB based on high yield losses caused by MCB damage. However, both parental lines are susceptible because they show a lot of MCB damage measured as tunnel length produced by MCB.

However, both parental lines are susceptible because they show large tunnel lengths by MCB attack. Each F_6_ was derived from a different F_2_ plant by using the single-seed descendant method (Butrón et al., 1998; Samayoa et al., 2014).

The phenotypic data for tunnel length produced by MCB and grain yield at 140g of H_2_O · kg^-1^, obtained by Samayoa et al. (2014), were used to carry out the phenotypic selection in the (A637 x EP42) RIL populations (original cycle, C0) and, along with genotyping data, to calculate the GEBV that were the basis of genomic selection.

The hundred forty-four RILs were previously genotyped with 130 polymorphic SSRs (Samayoa et al., 2014). These markers were relevant and informative and served to create a linkage map using a LOD threshold of two to declare significantly linked two markers, and a maximum distance of 50 cM among adjacent markers(Samayoa et al., 2014). GEBV for tunnel length and grain yield were estimated *via* ridge regression-BLUP (RR-BLUP) following Bernardo and Yu (2007). The performance of the RILs was modeled as:


y= μ1 + Xg + e

where y is an 144 × 1 vector of RIL phenotypic means (tunnel length, grain yield); μ is the overall mean of the 144 RILs; 1 is an 144 × 1 vector with all elements equal to 1; X is a 144×130 matrix with elements equal to 1 or -1 if RIL is homozygous for SSR allele from A637 (first parental inbred) or from EP42 (second parental inbred), respectively; g is an 130 × 1 breeding value vector associated with the SSR allele from A637 at each of the SSR loci and e is a vector (144 × 1) of residual effects.

The RILs evaluation trials carried out by Samayoa et al. (2014) were used to estimate the GV (genetic variance) and EV (environmental variance) in the original cycle C0. The variance of breeding values at each of the SSR loci was assumed equal to GV/SSR Number. GEBV (g) were obtained by solving the mixed-model equations being μ a fixed effect and g a random effect. SAS/IML software was used for the calculations (SAS 9.4, SAS Institute 2016).

Phenotypic data were used for developing three independent selection programs with three different selection criteria: 1) highest resistance (lower tunnels lengths produced by MCB) (PS-tunlen); 2) highest grain yield (PS-yield); and 3) the most resistant lines among those with a yield above the average (PS-index). For each selection program 20 RILs were chosen (selection intensity of 14%) and recombined to constitute the cycle 1 of selection. On the other hand, the estimated GEBVs were used to carry out three independent genomic selection programs using the same selection criteria and selection intensity than in phenotypic selection: GS-tunlen, GS-yield and GS-index. Three groups of 20 RILs were randomly selected for checking for genetic drift effects. Randomly selected RILs were recombined to constitute the RandomC1.

The recombination in each of the populations were carried out in 2013 by sowing a balanced number of grains from each of the constituent RILs and making plant-to-plant crosses, where each plant is involved in only one cross, either as a male or as female. In addition, the original cycle (C0) was reconstructed by recombining the 144 RILs using a 500 kernel-weighted mixture. In 2014, a second recombination of the C0 and the nine C1 populations was carried out.

In 2015, 150 plants from each C1 population were sown and self-fertilized, obtaining about 100 S_1_ families from each population. For phenotypic selection self -fertilized plants were infested with approx. 40 MCB eggs per plant deposited between the stem and the sheath of one basal leaf. Eggs reared at the MBG were used as described by Eizaguirre and Albajes (1992) and Khan and Saxena (1997). At harvest, the length of the tunnels produced by MCB were measured, the ears were collected and the yield per plant was evaluated using a subjective scale from 1=poor yield to 9=very good yield. The best 20 families from each population were selected and recombined to constitute the second PS cycles. On the other hand, for genomic selection, S_1_ families from GS-tunlenC1, GS-yieldC1 and GS-indexC1 were genotyped for the 130 SSRs used in the first cycle of genomic selection. The 20 best S_1_ families from each population families were selected based on their predicted breeding values, that is, Zg, where Z was an N × M matrix that corresponded to the genotypes for M SSR of the N families evaluated in C1s. The elements in Z were zero if the plants in C1 were heterozygous at the corresponding SSR loci. Selected families were recombined to constitute the second GS cycles. Families were randomly selected and recombined to form the RandomC2 cycle. The recombinations were performed in the same way as those that led to the first selection cycles (**Figure S1**).

In 2017, the first and second cycles of the 9 selections (3 PS, 3 GS and 3 random) were multiplied to obtain homogeneous seeds for the evaluations. In 2018 and 2019, the selection cycles were evaluated together with the original population (C0) at Pontecaldelas (42°23’31.4 “N 8°30’14.3 “W) and Barrantes (42°30’16.1 “N 8°46’16.2 “W) under natural infestation. Both locations during the two years showed a high intensity of MCB attack estimated as the averaged tunnel length across genotypes in each environment (44.3, 46.5, 21.8 and 10.3 cm in Barrantes 2018, Pontecaldelas 2018, Barrantes 2019 and Pontecaldelas 2019, respectively). Randomized complete block designs with three replications were used. Each experimental plot consisted of two rows of 25 plants · row^-1^ with a distance between rows of 0.8 m and between plants of 0.18 m. Data were recorded for grain moisture (percentage of water in total grain weight at harvest), yield (plot kernel weight at 14 kernel moisture referred in Mg · ha^-1^) and length of pith stem tunnels produced by MCB. In 2020, evaluations were also carried out at the Misión Biológica de Galicia (MBG) in Pontevedra (42°24’20.0 “N 8°38’41.9 “W) under artificial infestation and under insecticide protection. Eggs reared at the MBG as described by Eizaguirre and Albajes (1992) and Khan and Saxena (1997) were used to infest all plants in one row of the plot using about 40 eggs that were placed between the stem and the sheath of a basal leaf.

Data were analyzed with the Proc Mixed procedure of SAS software (SAS 9.4, SAS Institute 2016). Genotype was considered a fixed factor while replicates, environments (year-location-infestation condition combinations) and interactions between factors were considered random factors. Means were calculated for each genotype by the least squares procedure and compared by Fisher’s protected Least Significant Difference (LSD) at the 0.05 probability level. Finally, data for each trait were adjusted to linear regression models using a repeated measurement approach. Cycles was the independent variable; selection programs [each method (GS, PS or Random) x selection criteria (yield, tunnel length or index) combination] and the interaction cycle x selection programs were stablished as fixed effects and environments, repetitions and their interaction as random effects.

## 3 Results

**Figure 1:**
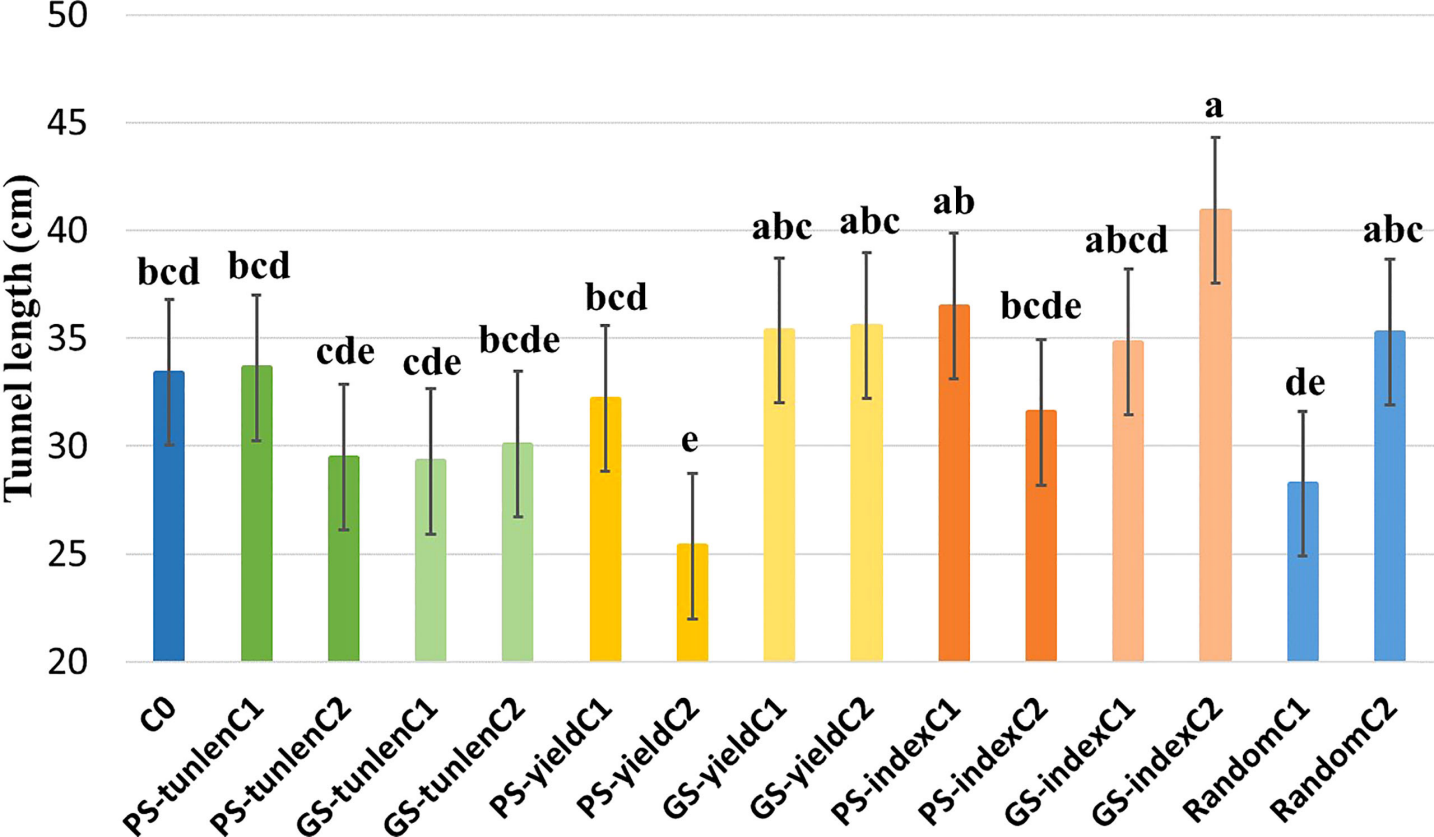
Comparison of means for tunnel length (cm) produced by MCB of the original cycle (C0);cycles of PS and GS to reduce tunnel length (tunlen); to improve grain yield (yield) and to reduce tunnel length while maintaining yield (index) and random selection cycles. Different letters indicate that means differed significantly at the 0.05 probability level.

A combined analysis over five environments was performed for tunnel length (the trial protected with insecticide was eliminated because no damage caused by MCB was observed). The genotype x environment interaction was not significant either for grain yield or tunnel length (**Table 1**). There were significant differences between the cycles within selection methods for the tunnel length produced by MCB. PS-yieldC2 and GS-indexC2 were the only ones that differed significantly (p< 0.05) from the original population. PS-yieldC2 was significantly more resistant than the original cycle but neither both cycles of genomic selection for tunnel length (GS-tunlenC1 and GS-tunlenC2) nor cycle 2 of phenotypic selection for tunnel length (PS-tunlenC2) differed from it. On the other hand, GS-indexC2 was the most susceptible selection cycle as it showed the largest tunnel. It should also be noted that there was no significant difference between the initial cycle and random selection; therefore, the effect of genetic random drift seems negligible for length of tunnels produced by MCB (**Figure 1**).

**Table 1:** Analysis of variance of the cycles of selection obtained by phenotypic and genomic selections for yield, tunnel length or an index that includes both traits.

Source of variation	Tunnel length		Yield	
	Z value	Pr > Z	Z value	Pr > Z
Environment (E)	1.38	0.0835	1.46	0.0727
Replication (E)	1.58	0.0572	1.33	0.0921
E x Cycle	0	1	0	1
Residual	9.9	< 0.0001	10.86	< 0.0001
	F value	Pr > F	F value	Pr > F
Cycle	2.68	0.0045	4.14	< 0.0001

A combined analysis on six environments was also performed for grain yield. There were significant differences between genotypes while the differences were not significant for the interaction (**Table 1**). The cycle with the highest yield was the second cycle of the GS for yield (GS-yieldC2) but it did not differ significantly from the second cycle of the PS to reduce tunnels (PS-tunlenC2) nor from the original cycle.

**Figure 2:**
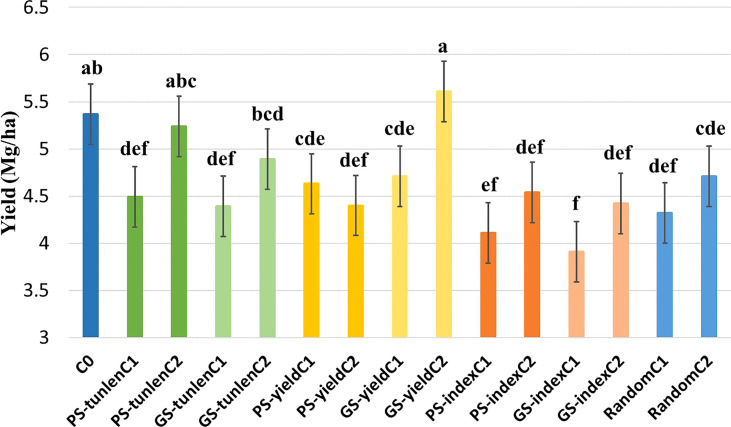
Comparison of grain yield (Mg·ha^-1^) of the original cycle (C0); cycles of PS and GS to reduce tunnel length (tunlen); to improve grain yield (yield) and to reduce tunnel length while maintaining yield (index) and random selection cycles. Different letters indicate that means differed significantly at the 0.05 probability level.

Significant difference for yield between the original cycle and the random selection indicate that the effect of genetic drift was important during the first cycle of selection, and therefore the different selection cycles will be compared to the first cycle of random selection to eliminate as much as possible genetic drift effects. Both GS-yieldC2 and PS-tunlenC2 differed significantly from randomC1 for yield (**Figure 2**).

**Figure 3:**
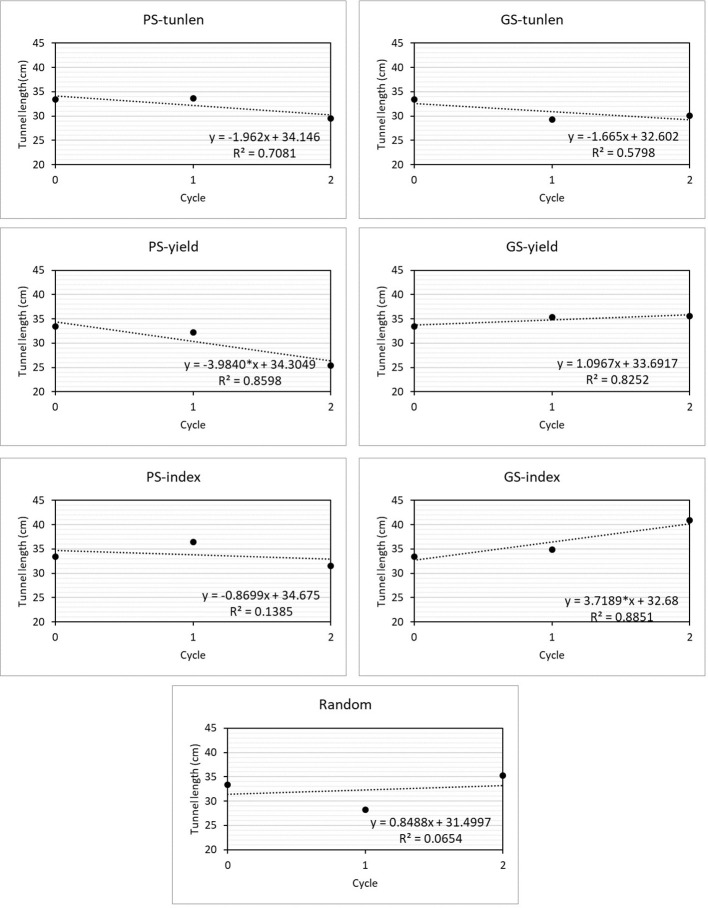
Linear regressions analysis for length of tunnels produced by MCB n selection cycles in six selection programs and one random selection. The linear response equation is shown for each of the selections, indicating whether the linear coefficient was significant at 0.05 (*).

Linear regressions allow evaluating how a trait varies throughout the cycles of a selection program. **Figure 3** shows the linear regressions of the length of tunnels produced by MCB on cycles of selection in seven selection programs. The regression coefficient was significantly different from zero in two selection programs: 1) phenotypic selection to improve yield (PS-yield) and GS to improve resistance while maintaining yield (GS-index). In the first case, the tunnels are reduced as the selection program progresses (b= -3.98) while in GS-index the tunnel lenght increase as the selection progresses (b=3.72). The best linear response to reduce tunnel length was obtained with PS-yield, but it did not differ significantly from those achieved with PS and GS for tunnel length (p = -0.34 for PS-yield *vs.* PS-tunlen b coefficients and p = 0.28 for PS-yield *vs.* GS-tunlen b coefficients) and PS-index (p = 0.15 for b coefficient comparison between PS-yield and PS-index).

**Figure 4:**
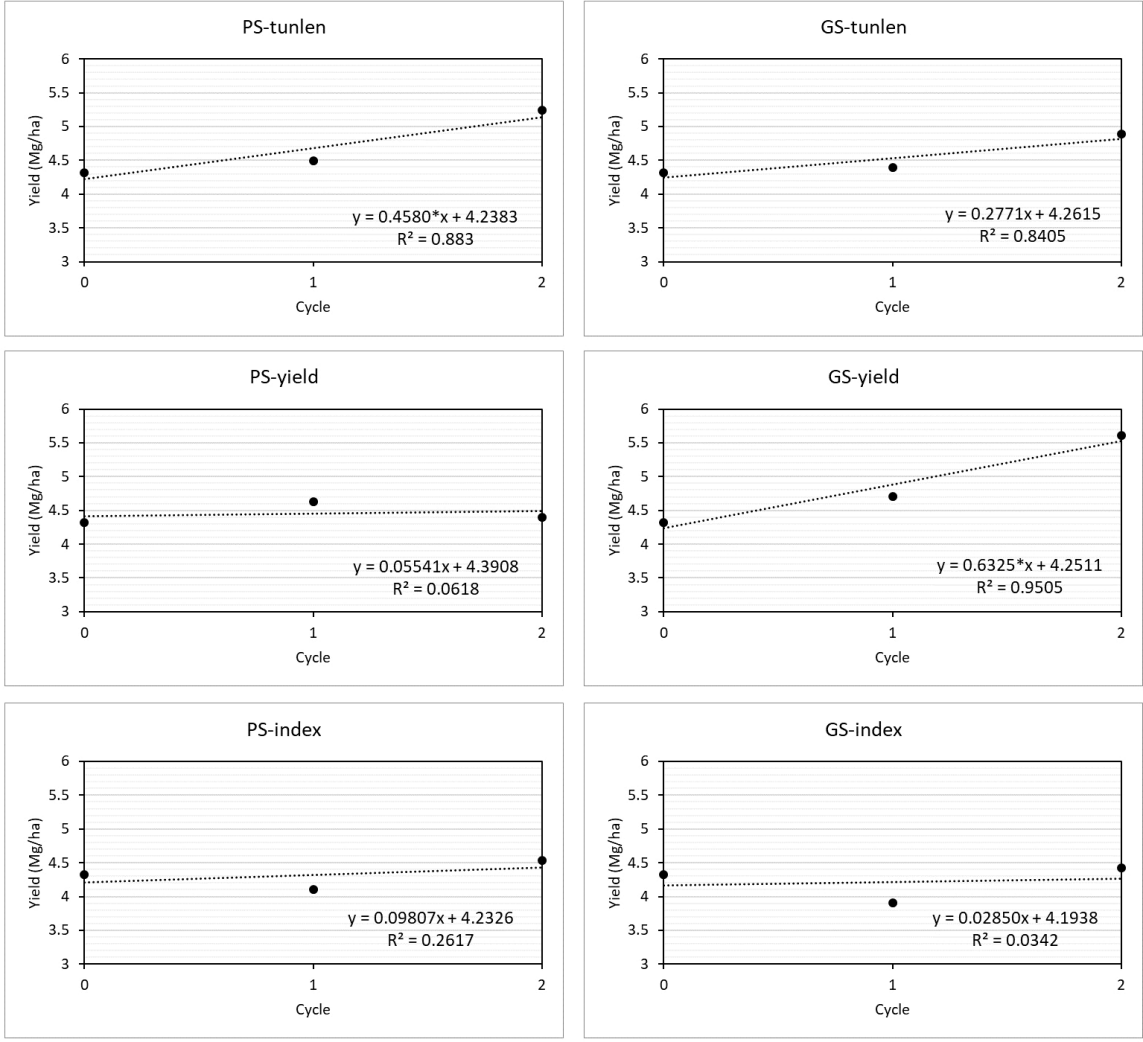
Linear regression analysis for yield (Mg·ha^-1^) of the selection cycles in six selection programs. The linear response equation is shown for each of the selections, indicating whether the linear coefficient was significant at 0.05 (*). RandomC1 was considered as original cycle (C0).

The selection programs GS-yield and PS-tunlen showed linear coefficients significantly different from zero (**Figure 4**). The best linear response was obtained with GS-yield (0.63), but it did not differ significantly from those achieved with PS-tunlen (p = 0.42) and GS-tunlen (p = 0.42).

## 4 Discussion

Two decades of research on maize resistance to MCB has demonstrated the high negative genetic correlation between yield potential and resistance. Such that pursuing higher resistance, measured as reduced tunnel length made by MCB in the pith, has significantly contributed to reduced kernel yield (Ordás et al., 2010; Butrón et al., 2012; Jiménez-Galindo et al., 2017; Samayoa et al., 2019). Although this negative relationship is not found in all genetic backgrounds (Samayoa et al., 2014; Jiménez-Galindo et al., 2019).

Some studies show that the inheritance of resistance to stem tunneling is basically under additive control by observing that improvement achieved through inbreeding is generally transmitted to hybrids (Butrón et al., 1999; Cartea et al., 1999). However, genotype differences for tunnel length can be biased by MCB genotype and MCB genotype × environment differences among plots, so the experimental error associated to tunnel length is high and makes that heritability for tunnel length is often low (Samayoa et al., 2014; Jiménez-Galindo et al., 2017). Moreover, resistance to MCB is a highly polygenic trait, where many genes are involved with small effects that are often undetected in QTL studies (Ordás et al., 2010; Samayoa et al., 2014; Samayoa et al., 2015; Jiménez-Galindo et al., 2017; Samayoa et al., 2019). The GS strategy based on genome-wide polymorphic markers that can capture minor gene effects on complex traits is presented as a possible alternative to phenotype and marker-assisted selections (Heffner et al., 2010; Liu et al., 2018; Rice and Lipka, 2021). Our results indicate that the GS-tunlen C1 and C2 and PS-tunlenC2 cycles did not differ from the cycle with the shortest tunnels (PS-yieldC2), confirming that genetic variability for resistance to stem tunneling can be managed by phenotypic and genomic selections, although the gains did not differ significantly from zero. Although the additive component was the most important for tunnel length among generations derived from A637 × EP42 (Butrón et al., 2009), heritability in the (A637 × EP42) F_2_ population was low, *h^2 ^= *0.34 (Samayoa et al., 2014) which could explain the lack of a significant improvement by PS. GS was also ineffective to significantly reduce tunnel length likely due to the fact that genotype predictions are often not highly accurate when non-genetic factors are a major source of trait variation as it is the case for tunnel length due to a high experimental error (Hickey et al., 2017). However, genomic prediction accuracy could be improved by increasing the number of markers because less than 200 markers could be enough to achieve the highest prediction accuracy for traits with moderate heritability in F_2_ and backcrossing populations (Lorenzana and Bernardo, 2009; Lian et al., 2014; Zhang et al., 2015), but Hao et al. (2019) estimated that the highest accuracy was achieved with 800 SNPs when genomic selection is performed in a RIL population because linkage disequilibrium is lower among RILs compared to F_2_ and backcrossing mapping populations.

PS-yieldC2 was the only selection scheme that significantly reduced tunnel length compared to the original cycle. Phenotypic selection for yield could improve resistance because, in this material, the genetic correlation between resistance and yield seems important and positive, contrary to other genetic backgrounds where this correlation is negative (Butrón et al., 2012). Another aspect that could contribute to the success of PS-yield for reducing tunnel length is the method used to select the S_1_ families that formed the PS-yieldC2. The ears were not weighed because they come from self-pollinated plants and the amount of grain depends on the moment of pollination; instead, ear yield was assessed by a subjective scale and good looking ears would probably come from healthier plants, tunnels by MCB being determinant in plant health.

Inheritance of yield has an important dominant component, which could lead to a clear inbreeding depression effect when few individuals are selected in a highly variable population. The yield of RandomC1 was significantly lower than that of the original population (4.32 *vs.* 5.37 ± 0.38 Mg·ha^-1^) confirming that the selection scheme used generated genetic drift that, in turn, originated inbreeding depression on yield. Therefore, in order to correct for the effect of genetic drift on yield, the RandomC1 cycle was used as the starting point to calculate yield response to selection. GS-yield was the most effective method for increasing yield; meanwhile PS was ineffective to improve the yield. Given that the inheritance of the yield has an important dominant component, the best phenotypes could be those with more favorable allelic interactions for yield. While GS was based on additive variation, which is the heritable genetic component, and was much more effective (Liu et al., 2018). In this work, it has been verified, as stated before by other authors (Zhang et al., 2017; Rice and Lipka, 2021), that GS is a powerful tool for improving the yield of maize.

Selections using indices, both GS and PS, have not been successful in improving resistance to MCB while maintaining yield. Indeed, GS for index values attained a significant increase of tunnels after two cycles of selection and no changes were observed in yield after GS and PS selections. Phenotypic selection for index values did not get a significant gain for tunnel length contrarily to index selection made previously in a maize composite named EPS12 (Sandoya et al., 2008; Butrón et al., 2012). These authors reported that the length of the tunnels and yield had been significantly reduced using the same index selection approach. The lower response to index phenotypic selection observed in the current study would likely be related to the narrowed variability for resistance to MCB in the (A637 × EP42) F2 population compared to the EPS12 composite since A637 and EP42 were both susceptible to MCB attack (Butrón et al., 1998).

In summary, the linear response of yield to GS-yield did not differ significantly from responses to GS and PS selections for tunnel length. Similarly, PS-yield selection, which showed the highest tunnel length reduction, presented a linear response on tunnel length that did not differ significantly from those obtained by GS and PS selections. Suggesting that phenotypic or genotypic selection for tunnel length may be more effective in improving both traits, but the gains per cycle would be small for both traits. However, from a practical point of view, high yield, regardless of the damage caused by MCB, would be the most valuable trait by farmers and genomic selection for yield has been proven as the most successful methodology to increase yield.

## Data availability statement

The original contributions presented in the study are included in the article/**Supplementary Material**. Further inquiries can be directed to the corresponding author.

## Author contributions

RM and BO conceived the study and performed selection programs with the assistance of AB, JJ-G and LS. MF coordinated and carried out the genotyping work to carry out the genomic selection with the collaboration of AC. RM, JJ-G, LS, and AB took care of field experiments, data recording, and sample collection. RM performed statistical analyses of data with the assistance of NG. NG drafted and edited the manuscript. All authors have read and approved the final version of the manuscript. All authors contributed to the article.

## Supplementary material

Supplementary Figure 1 Summary figure of breeding programs based on phenotypic or genotypic values for a single trait (yield or MCB susceptibility measured as tunnel length) or both traits (index).
