# Supplementary figures and images for: Genomic versus phenotypic selection to improve corn borer resistance and grain yield in maize
Source: Front Plant Sci. 2023 Jul 7;14:1162440. doi: 10.3389/fpls.2023.1162440 (PMC10360656; doi:10.3389/fpls.2023.1162440)

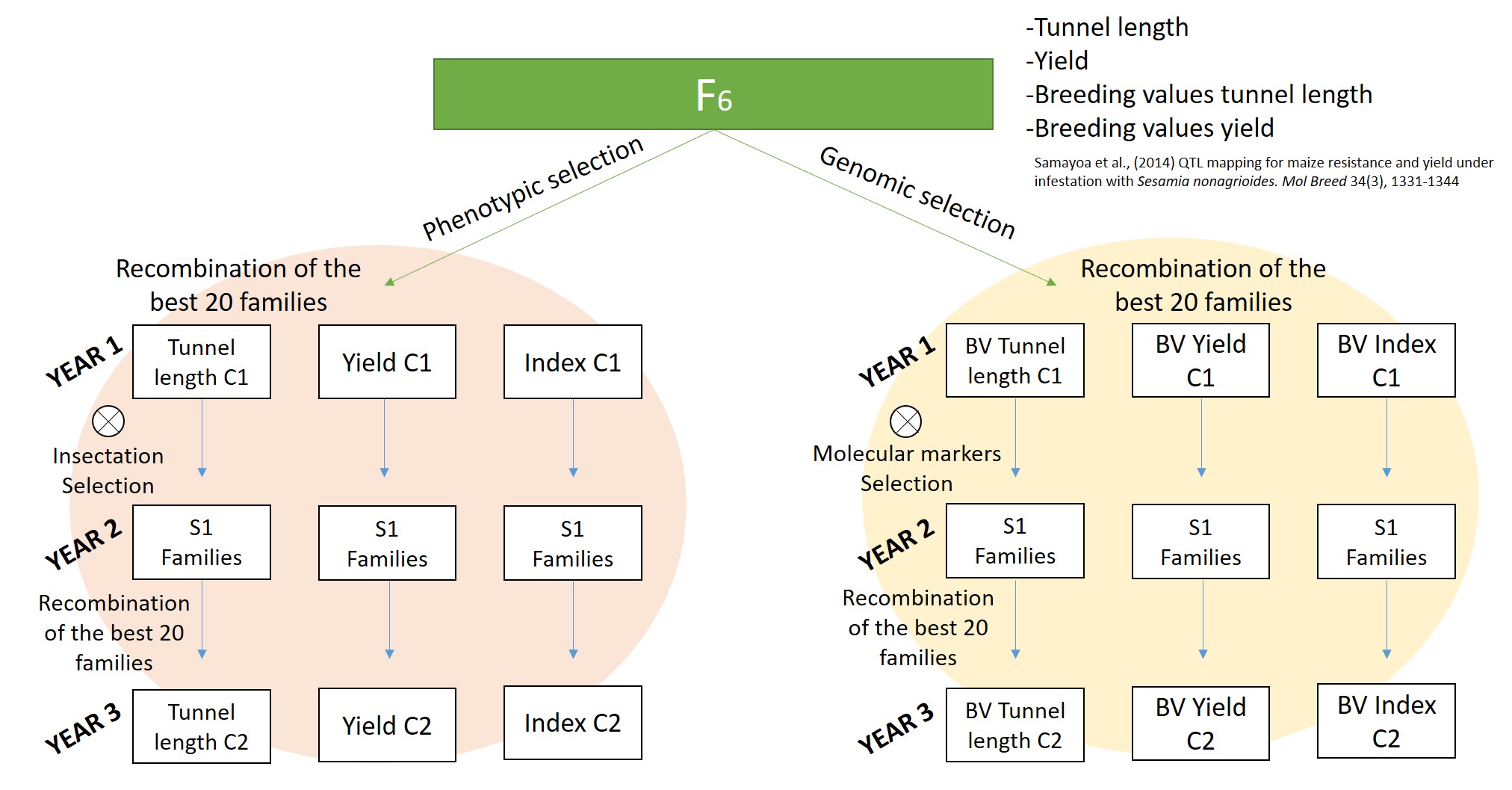

Supplement: Supplementary Figure 1 — Summary figure of breeding programs based on phenotypic or genotypic values for a single trait (yield or MCB susceptibility measured as tunnel length) or both traits (index). [file Image_1.jpg]
